# Supplementary material for: Investigating the Relationship Between Ultra-Processed Food Consumption and Academic Performance in the Adolescent Population: The EHDLA Study
Source: Nutrients. 2025 Jan 31;17(3):524. doi: 10.3390/nu17030524 (PMC11819819; doi:10.3390/nu17030524)

Table S1. Complete list of ultra-processed food items and their groupings from the food frequency questionnaire.

Category	Included items
Overall UPF	Item 3. Chocolate (bars, bonbons, "Kit Kat", "Mars", etc.).<br>Item 4. Breakfast cereals ("Corn-Flakes", "Kellogg's").<br>Item 5. Plain biscuits (e.g., "Maria" cookies).<br>Item 6. Chocolate or cream-filled biscuits.<br>Item 7. Muffins, sponge cake, etc.<br>Item 8. Puff pastry, donuts, croissants, etc.<br>Item 16. Soups and creams.<br>Item 24. Croquettes, *empanadas*, pizza.<br>Item 20. Minced meat, sausage, hamburgers.<br>Item 26. Cured ham, sweet ham, sausages.<br>Item 33. Commercial fruit juices.<br>Item 35. Dairy desserts (custards, flan, cottage cheese).<br>Item 36. Cream or chocolate cakes.<br>Item 37. Sweets (gummies, candies, caramels, etc.).<br>Item 38. Packaged snacks ("chips", "Cheetos", "fried snacks", etc.).<br>Item 39. Ice cream.<br>Item 40. Sugary drinks ("Coca-Cola", "Fanta", etc.).<br>Item 41. Low-calorie beverages ("Coca-Cola Light", "diet drinks", etc.).<br>Item 44. Distilled drinks (whiskey, gin, cognac, etc.).
Individual	
Sausages	Item 20. Minced meat, sausage, hamburgers.<br>Item 26. Cured ham, sweet ham, sausages.
Fast-food	Item 16. Soups and creams.<br>Item 24. Croquettes, *empanadas*, pizza.
Dairy products	Item 35. Dairy desserts (custards, flan, cottage cheese).<br>Item 36. Cream or chocolate cakes.<br>Item 39. Ice cream.
Beverages	Item 33. Commercial fruit juices.<br>Item 40. Sugary drinks ("Coca-Cola", "Fanta", etc.).<br>Item 41. Low-calorie beverages ("Coca-Cola Light", "diet drinks", etc.).<br>Item 44. Distilled drinks (whiskey, gin, cognac, etc.).
Fried foods	Item 38. Packaged snacks ("chips", "Cheetos", "fried snacks", etc.).
Sweets	Item 3. Chocolate (bars, bonbons, "Kit Kat", "Mars", etc.).<br>Item 4. Breakfast cereals ("Corn-Flakes", "Kellogg's").<br>Item 5. Plain biscuits (e.g., "Maria" cookies).<br>Item 6. Chocolate or cream-filled biscuits.<br>Item 7. Muffins, sponge cake, etc.<br>Item 8. Puff pastry, donuts, croissants, etc.<br>Item 37. Sweets (gummies, candies, caramels, etc.).

FFQ, food frequency questionnaire; UPF, ultra-processed food.

Table S2. Generalized linear model examining the association of servings of ultra-processed food consumed (and covariates) with academic performance (grade point average) among adolescents.

Predictors	*B*	95% CI	*p*-value
UPF (per one serving)	-0.58	-0.86, -0.31	<0.001
Age (per one year)	-0.09	-0.20, 0.01	0.092
Sex			
Boys	Reference		
Girls	0.50	0.19, 0.82	0.002
FAS-III (per one point)	0.16	0.09, 0.24	<0.001
BMI (per one kg/m^2)	-0.02	-0.05, 0.02	0.301
Overall sleep duration (per one hour)	9.51	-1.43, 20.46	0.089
YAP-S physical activity (per one point)	0.13	-0.11, 0.36	0.288
YAP-S sedentary behaviors (per one point)	-0.18	-0.45, 0.09	0.184

B, unstandardized beta coefficient; CI, confidence interval; UPF, ultra-processed food.

Table S3. Generalized linear model examining the association of servings of ultra-processed food consumed (and covariates) with academic performance in language among adolescents.

Predictors	*B*	95% CI	*p*-value
UPF (per one serving)	-0.56	-0.86, -0.26	<0.001
Age (per one year)	-0.10	-0.21, 0.02	0.105
Sex			
Boys	Reference		
Girls	0.81	0.46, 1.15	<0.001
FAS-III (per one point)	0.15	0.07, 0.23	<0.001
BMI (per one kg/m^2)	0.00	-0.04, 0.03	0.842
Overall sleep duration (per one hour)	7.08	-4.96, 19.12	0.250
YAP-S physical activity (per one point)	0.22	-0.04, 0.48	0.096
YAP-S sedentary behaviors (per one point)	-0.22	-0.51, 0.08	0.148

B, unstandardized beta coefficient; CI, confidence interval; UPF, ultra-processed food.

Table S4. Generalized linear model examining the association of servings of ultra-processed food consumed (and covariates) with academic performance in math among adolescents.

**Predictors**	***B***	**95% CI**	***p*-value**
UPF (per one serving)	-0.58	-0.92, -0.25	<0.001
Age (per one year)	-0.11	-0.24, 0.02	0.098
Sex			
Boys	Reference		
Girls	0.21	-0.17, 0.60	0.278
FAS-III (per one point)	0.18	0.10, 0.27	<0.001
BMI (per one kg/m^2)	-0.03	-0.07, 0.01	0.216
Overall sleep duration (per one hour)	9.28	-4.08, 22.63	0.174
YAP-S physical activity (per one point)	0.09	-0.19, 0.38	0.525
YAP-S sedentary behaviors (per one point)	-0.26	-0.59, 0.06	0.115

B, unstandardized beta coefficient; CI, confidence interval; UPF, ultra-processed food.

Table S5. Generalized linear model examining the association of servings of ultra-processed food consumed (and covariates) with academic performance in English among adolescents.

**Predictors**	***B***	**95% CI**	***p*-value**
UPF (per one serving)	-0.61	-0.90, -0.32	<0.001
Age (per one year)	-0.07	-0.18, 0.04	0.199
Sex			
Boys	Reference		
Girls	0.50	0.17, 0.83	0.003
FAS-III (per one point)	0.15	0.07, 0.22	<0.001
BMI (per one kg/m^2)	-0.02	-0.06, 0.01	0.181
Overall sleep duration (per one hour)	12.01	0.44, 23.58	0.042
YAP-S physical activity (per one point)	0.14	-0.10, 0.39	0.250
YAP-S sedentary behaviors (per one point)	-0.05	-0.34, 0.23	0.712

B, unstandardized beta coefficient; CI, confidence interval; UPF, ultra-processed food.

Table S6. Generalized linear models examining the association of servings of different ultra-processed food groups consumed (and covariates) with academic performance (grade point average) among adolescents.

Predictors	*B*	95% CI	*p*-value
Sausages (per one serving)	-0.02	-0.05, 0.01	0.287
Fast-food (per one serving)	-0.05	-0.09, -0.01	0.010
Dairy products (per one serving)	-0.05	-0.08, -0.02	<0.001
Beverages (per one serving)	-0.07	-0.10, -0.04	<0.001
Fried foods (per one serving)	-0.10	-0.16, -0.04	0.002
Sweets (per one serving)	-0.02	-0.03, -0.01	0.001

B, unstandardized beta coefficient; CI, confidence interval. Adjusted for sex, age, body mass index, physical activity, sedentary behavior, and sleep duration.

Figure S1. Estimated marginal means of different academic performance indicators based on ultra-processed food status among adolescents. GPA, grade point average (i.e., the mean of all academic subject scores for each student); UPF, ultra-processed food.

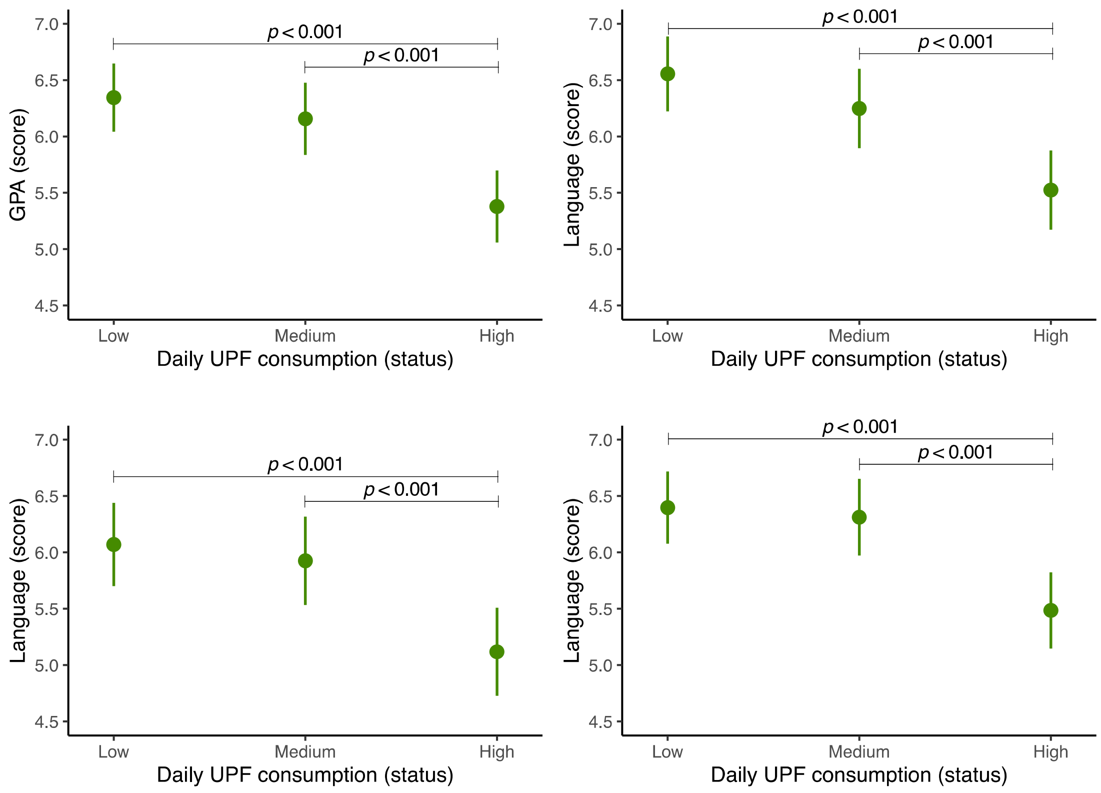

Supplement: Supplementary file 1 [file nutrients-17-00524-s001.zip › nutrients-3459854-supplementary.pdf]
